# Fatigue Reliability Assessment of RC Beams in Heavy-Haul Railways Based on Point Estimate Method

**DOI:** 10.3390/ma16227098

**Published:** 2023-11-09

**Authors:** Jiarui Shi, Li Song, Chenxing Cui, Zhiwu Yu

**Affiliations:** 1 School of Civil Engineering, Central South University, Changsha 410075, China; shijiarui1995@163.com (J.S.); songli@csu.edu.cn (L.S.); zhwyu@csu.edu.cn (Z.Y.); 2 National Engineering Research Center of High-Speed Railway Construction Technology, Changsha 410075, China; 3 School of Civil Engineering, Henan University of Technology, Zhengzhou 450001, China

**Keywords:** heavy-haul railways, reinforced concrete beam, point estimate method, equivalent stress range, fatigue reliability, failure probability

## Abstract

Heavy-haul railways have a high passing frequency of trains with a large axle weight, causing rapid accumulation of fatigue damage in reinforced concrete (RC) bridge structures, which significantly affects the safety of the bridges. To study the fatigue reliability of RC beams in heavy-haul railways, the fatigue performance function for RC beams in heavy-haul railways was established, and the fatigue reliability assessment method for bridge structures in heavy-haul railways based on the point estimate method (PEM) was developed. An 8 meter-span plate beam in an existing heavy-haul railway illustrates the method. The train axle weight and dynamic coefficient were considered random variables, and the first four moments of equivalent stress ranges were obtained. The traffic quantity of the heavy-haul railways was investigated, and the fatigue reliability was evaluated using the proposed method. In addition, the effects of annual freight volume and train axle weight on fatigue reliability were discussed. Results indicate that PEM can effectively and accurately evaluate the fatigue reliability of RC beams in heavy-haul railways. In the first 20 years of operation, the fatigue failure probability was less than the limit value specified in the standard. The increase in annual traffic volume and train axle weight will cause a significant increase in fatigue failure probability. The research results of this paper are expected to provide an important basis for the design and maintenance of reinforced concrete bridges for heavy-haul railways in the future.

## 1. Introduction

Heavy-haul railways have a large traction weight and high transportation efficiency, which can bring huge economic and extensive social benefits, and have become one of the main development fields of modern rail transit [1,2,3]. As the axle load and traffic frequency of heavy-haul railway trains gradually increase, the transportation efficiency of heavy-haul railways will continue to improve, but the structural service performance will deteriorate [4,5]. The bridge structure rarely undergoes damage in the early stages of operation, and the safety issues of the bridge mainly include service life and residual strength [6,7,8]. The reinforced concrete (RC) structure is the most commonly used structural form for heavy-haul railway bridges. During the operation of heavy-haul railway RC bridges, they are subjected to repeated long-term train loads, leading to a continuous accumulation of fatigue damage in both the steel and concrete components within the structure. Fatigue damage is one of the main reasons for the deterioration of RC beams [9,10,11]. The increase in train axle load and annual traffic volume will increase the risk of fatigue cracking and failure of RC bridges. During the actual operation of bridges, both material parameters and train loads are random variables, and there is a certain risk of failure during the operation of bridge structures. Therefore, conducting a fatigue reliability assessment of RC beams for heavy-haul railways is of great significance for ensuring the safe service of heavy-haul railway bridges.

Currently, research on the fatigue performance of RC beams for heavy-haul railways focuses on testing and fatigue life assessment. Yu et al. [12] used a multi-sensor system to monitor the performance degradation of a scaled model of a typical heavy-haul railway bridge under fatigue load and proposed a damage evolution model that considers the coupling of specimen stiffness degradation and inelastic deformation. Li et al. [13,14,15] conducted fatigue tests on large-scale specimens of heavy-haul railway bridges, and obtained the fatigue failure morphology, fatigue damage evolution law, and static bearing performance after the fatigue of heavy-haul railway bridges. Song and Yu [16] conducted fatigue performance tests on prestressed concrete beams of heavy-duty railways using fiber optic grating sensing technology. Lu et al. [17] conducted fatigue performance tests on 8 m low-height RC plate beams of heavy-haul railways; obtained the fatigue failure modes of the plate beams; and obtained the development patterns of beam cracks, stiffness, and strain with the number of fatigue load cycles. Cui et al. [18] conducted fatigue damage analysis and life assessment on 8 m RC beams of the Daqin Heavy Haul Railway and studied the impact of annual transportation volume on fatigue life. Wei et al. [19] established a time-varying model for the stiffness of heavy-haul railway bridges and evaluated their service performance. At present, there has been extensive research on the adaptability of static, dynamic, and fatigue performance of railway bridges with the increase in train axle load and traffic frequency [20,21,22,23,24], while there is relatively little research on the fatigue reliability evaluation of heavy-haul railway bridges. Extensive research has also been conducted on the fatigue reliability of reinforced concrete beams [25,26,27,28,29], but existing research mainly focuses on Monte Carlo methods. The Monte Carlo method is a numerical simulation method that takes probability phenomena as the research object. This method is suitable for problems that cannot be solved directly using analytical methods or are difficult to solve. The central idea of the Monte Carlo method is that in order to obtain a solution to the problem, a random process or probability model should be established first, and then the failure probability should be obtained by observing or sampling the random process or probability model. When the number of sampling times of the Monte Carlo method is infinite, the simulation results tend to approach the exact solution, but the disadvantage is that the computational workload is often huge, even unacceptable.

Based on the operational characteristics of heavy-haul railways, this article establishes a fatigue reliability assessment framework for RC beams in heavy-haul railway infrastructure. It further develops a fatigue reliability evaluation method for heavy-haul railway bridge structures based on the point estimation approach and applies it to an 8 m RC plate beam in a specific heavy-haul railway system. The train load model of the heavy-haul railway was analyzed, and the historical equivalent operating spectrum of the heavy-haul railway was calculated. The first four moments of the equivalent stress range were obtained for the unknown probability distribution type by considering train axle load and dynamic coefficient as random variables. Subsequently, fatigue reliability evaluation was conducted on the plate beam based on this analysis, exploring the impact of train axle load and annual traffic volume on fatigue reliability. The research results of this paper are expected to provide an important basis for the design and maintenance of reinforced concrete bridges for heavy-haul railways in the future.

## 2. The Fatigue Performance Function for RC Beams in Heavy-Haul Railways

For heavy-haul railway RC beams, fatigue failure originates from the fatigue fracture of the steel bars at the bottom of the beam [16,17]. The S-N curve defines the relationship between the stress range of steel bars and the number of fatigue failure cycles (fatigue life) of steel bars, which can be expressed as [1]:

(1) lgN=lgC−mlg(Δσ)

where *N* is the number of fatigue failure cycles (fatigue life) of the steel bars; Δσ is the constant amplitude fatigue stress range of the steel bars; *C* and *m* are fatigue performance constants and material characteristic constants, respectively.

Under the action of train load, the steel bars inside the beam generate variable amplitude stress cycles. The linear damage accumulation criterion, also known as the Miner criterion, is commonly used for fatigue damage calculation under variable amplitude stress cycles [30]:

(2) $$D=\sum \frac{n_{i}}{N_{i}}=\frac{1}{C}\sum n_{i}{\left(\Delta \sigma_{i}\right)}^{m}$$

where *D* represents fatigue damage; *n_i_* is the number of cycles of stress range $\Delta \sigma_{i}$; *N_i_* is the number of cycles during fatigue failure of steel bars under stress range $\Delta \sigma_{i}$.

The equivalent stress range is commonly used to replace the various stress ranges of the variable amplitude stress cycle. The equivalent stress range is obtained according to the equivalent principle, that is, the number of cycles of the equivalent stress range and the fatigue damage caused by it are equal to the total number of cycles of the stress range of all the varying stress cycles and the fatigue damage caused by it. Therefore, the equivalent force amplitude *S*_re_ can be calculated as [31]:

(3) $$S_{\mathrm{re}}={\left(\sum \frac{n_{i}{\left(\Delta \sigma_{i}\right)}^{m}}{\sum n_{i}}\right)}^{1/m}$$

where $\sum n_{i}$ is the total number of cycles of variable amplitude stress cycling. The cumulative fatigue damage *D*_s_ of steel bars in a single train passing the bridge can be expressed as:

(4) $$D_{s}=\frac{S_{\mathrm{re}}^{m}{\sum n}_{i}}{C}$$

In the absence of maintenance, the accumulation of fatigue damage is continuous. When the railway operates *K* years, the cumulative fatigue damage *D*_t_ of steel bars can be calculated as:

(5) $$D_{t}=\sum_{j=1}^{J}\sum_{k=1}^{K}D_{s,j}f_{j,k}$$

where *D*_s,*j*_ is the fatigue damage to the steel bars caused by a single *j* type train passing through the bridge; *f_j_*_,*k*_ is the operating frequency of type *j* trains in the *k*-th year of heavy-haul railway operation; *J* is the total number of operating train types.

When the cumulative fatigue damage of steel bars defined by Formula (5) reaches a critical level, the fatigue failure of RC beams in heavy-haul railways will occur. The fatigue performance function of RC beams in heavy-haul railways can be defined as follows:

(6) $$G(x)=D_{c}-D_{t}$$

where *D*_c_ is the critical fatigue damage of the steel bars when fatigue fracture occurs, representing the fatigue resistance of the steel bars. Substituting Formulas (4) and (5) into the above equation, the fatigue performance function of heavy-haul railway RC beams can be transformed into:

(7) $$G(x)=D_{c}-\sum_{j=1}^{J}\sum_{k=1}^{K}\frac{S_{\mathrm{re},j}^{m}{\sum n}_{i,j}}{C}f_{j,k}$$

The critical fatigue damage *D*_c_, fatigue performance constant *C*, and equivalent stress range *S*_re_ are considered random variables in the fatigue function. Due to the small variability of the material characteristic constant *m*, its randomness may not be considered [32]. The train operating frequency *f_j_*_,*k*_ can be obtained from the operating spectrum of heavy-haul railways. When the fatigue performance function is greater than 0, the fatigue resistance is greater than the load effect, and the component is in a fatigue safe state.

## 3. Point Estimate Method for Structural Reliability Evaluation

### 3.1. Statistical Moment Estimation of the Performance Function

Zhao and Ono [33] developed a point estimate method for statistical moments of the performance function. For the performance function of structural response Z=G(x), the statistical moments of each order of the performance function can be calculated by the following formula:

(8) $$\left\{\begin{array}{l} \mu_{G}=\int G(x)f_{X}(x)dx\\ \begin{array}{cc} M_{zG}=\int {\left(G(x)-\mu_{G}\right)}^{z}\;f_{X}(x)dx, & z\geq 2 \end{array} \end{array}\right.$$

where $f_{X}(x)$ is the joint probability density function of n-dimensional random variables x; $\mu_{G}$ is the mean of the performance function Z=G(x); $M_{zG}$ is the *z*-order central moment of the performance function.

If each random variable is independent of each other, according to the inverse normal transformation of the random variable, the statistical moments of each order of the performance function can be expressed as:

(9) $$\left\{\begin{array}{l} \mu_{G}=\int G\left(T^{-1}(u)\right)\phi_{U}(u)du\\ \begin{array}{cc} M_{zG}=\int {\left(G\left(T^{-1}(u)\right)-\mu_{G}\right)}^{z}\;\phi_{U}(u)du, & z\geq 2 \end{array} \end{array}\right.$$

where ***u*** represents an *n*-dimensional standard normal random vector $u={\left(u_{1},\;u_{2},\;\cdots ,\;u_{n}\right)}^{T}$; $T^{-1}(u)$ represents the inverse normal transformation of random variables, and this paper adopts the fourth order moment inverse normal transformation [34]; $\phi_{U}(u)$ represents the joint probability distribution of *n*-dimensional standard normal random variable ***u***.

For simple performance functions, the statistical moments of each order can be directly integrated from the above equations. In engineering, performance functions are usually complex nonlinear functions or even implicit functions, leading to the statistical moments of each order of the functional function being difficult to be directly integrated and calculated. In this case, the dimensionality reduction integration method is usually used. In previous studies, the most commonly used methods were one-dimensional dimensionality reduction [33] and two-dimensional dimensionality reduction [35]. In this paper, the two-dimensional dimensionality reduction method is used, with $L(u)=G(T^{-1}(u))$. For *n*-dimensional random variables, Equation (9) can be transformed into the following formula:

(10) $$\left\{\begin{array}{l} \begin{array}{l} \mu_{G}\approx \sum_{p<q}E\left(L(u_{p},u_{q},u_{c})\right)\\ \;\;\;\;\;\;\;-(n-2)\sum_{l=1}^{n}E\left(L(u_{l},u_{c})\right)\\ \;\;\;\;\;\;\;+\frac{\left(n-1)(n-2\right)}{2}L(u_{c}) \end{array}\\ \begin{array}{l} M_{zG}\approx \sum_{p<q}E\left({\left(L(u_{p},u_{q},u_{c})-\mu_{G}\right)}^{z}\right)\\ \;\;\;\;\;\;\;\;\;-(n-2)\sum_{l=1}^{n}E\left({\left(L(u_{l},u_{c})-\mu_{G}\right)}^{z}\right)\\ \;\;\;\;\;\;\;\;\;+\frac{\left(n-1)(n-2\right)}{2}{\left(L(u_{c})-\mu_{G}\right)}^{z},\;\;\;z\geq 2 \end{array} \end{array}\right.$$

where $L(u_{c})$ is the response value of all standard normal random variables at the reference point, generally taking $u_{c}=0$; $L(u_{l},u_{c})$ represents the response values of all other variables at the reference point when the *l*-th variable is a random variable; $L(u_{p},u_{q},u_{c})$ represents the response values when the *p*-th and *q*-th variables are random variables; and all other variables are at the reference point.

Because all random variables in the equation $u={\left(u_{1},\;u_{2},\;\cdots ,\;u_{n}\right)}^{T}$ follow a standard normal distribution, $E({\left(L(u_{l},u_{c})-\mu_{G}\right)}^{z})$ and $E({\left(L(u_{p},u_{q},u_{c})-\mu_{G}\right)}^{z})$ can be calculated using the Gaussian–Hermite integral [33]:

(11) $$\left\{\begin{array}{l} \begin{array}{l} E\left({\left(L(u_{l},u_{c})-\mu_{G}\right)}^{z}\right)\\ \;\;=\sum_{i=1}^{h}P_{r,i}{\left(L(u_{r,i},u_{c})-\mu_{G}\right)}^{z} \end{array}\\ \begin{array}{l} E\left({\left(L(u_{p},u_{q},u_{c})-\mu_{G}\right)}^{z}\right)\\ \;\;=\sum_{i=1}^{h}\sum_{j=1}^{h}P_{r,i}P_{r,j}{\left(L\left(u_{r,i},u_{r,j},u_{c}\right)-\mu_{G}\right)}^{z} \end{array}\\ \begin{array}{cc} u_{r}=\sqrt{2}x_{\mathrm{GH}}, & P_{r}=\frac{w_{\mathrm{GH}}}{\sqrt{\pi }} \end{array} \end{array}\right.$$

where *h* is the estimated number of points and equal to the number of integration points for Gaussian–Hermite integration; *u*_r_ and *P*_r_ are the abscissa values and corresponding weight values of the estimated point, respectively; $x_{\mathrm{GH}}$ and $w_{\mathrm{GH}}$ are the horizontal coordinates and weight coefficients of the integration points for the Gaussian–Hermite integral, respectively. When *h* is taken as 5, the estimated points and corresponding weight values are shown in Table 1.

### 3.2. The Failure Probability of Performance Function

After obtaining the central moment of the performance function according to the point estimate method, the standard deviation $\sigma_{G}$, skewness $\alpha_{3G}$, and kurtosis $\alpha_{4G}$ of the performance function Z=G(x) can be calculated by the following formulas:

(12) $$\left\{\begin{array}{l} \sigma_{G}=\sqrt{M_{2G}}\\ \alpha_{3G}=M_{3G}/\sigma_{G}^{3}\\ \alpha_{4G}=M_{4G}/\sigma_{G}^{4} \end{array}\right.$$

The fourth moment reliability index $\beta_{4M}$ of the performance function can be calculated using the following formula [34]:

(13) $$\beta_{4M}=\frac{\sqrt[{3}]{2}p_{G}}{\sqrt[{3}]{-q_{G}+k_{G}}}-\frac{\sqrt[{3}]{-q_{G}+k_{G}}}{\sqrt[{3}]{2}}+\frac{b_{3G}}{3b_{4G}}$$

The coefficients in the formula are calculated by the following formula:

(14) $$\left\{\begin{array}{l} \begin{array}{cc} k_{G}=\sqrt{q_{G}^{2}+4p_{G}^{3}}, & p_{G}=\frac{3b_{2G}b_{4G}-b_{1G}^{2}}{9b_{4G}^{2}} \end{array}\\ q_{G}=\frac{2b_{3G}^{3}+9b_{1G}b_{2G}b_{4G}+27b_{4G}^{2}(b_{1G}+\mu_{G}/\sigma_{G})}{27b_{4G}^{3}}\\ b_{1G}=-b_{3G}=-\frac{\alpha_{3G}}{6(1+6\varsigma_{G})}\\ b_{2G}=\frac{1-3\varsigma_{G}}{1+b_{1G}^{2}-\varsigma_{G}^{2}},\;b_{4G}=\frac{\varsigma_{G}}{1+b_{1G}^{2}+12\varsigma_{G}^{2}}\\ \varsigma_{G}=\frac{1}{36}\left(\sqrt{6\alpha_{4G}-8\alpha_{3G}^{2}-14}-2\right) \end{array}\right.$$

Finally, the failure probability *P*_f_ of the performance function Z=G(x) can be calculated by the following formula:

(15) $$P_{f}=\Phi \left(-\beta_{4M}\right)$$

where Φ( ⋅ ) is the cumulative distribution function of the standard normal distribution.

## 4. Engineering Application

### 4.1. Project Overview

The engineering case under analysis pertains to a RC plate beam bridge situated on a heavy-haul railway in China, which has been operational for 20 years. The RC simply supported plate beam is one of the commonly used structures in small-span bridges of heavy-haul railways. The vertical stiffness of RC plate beams in heavy-haul railways is relatively low, and the issue of fatigue damage evolution and accumulation is prominent. An increase in axle load can also cause a serious decrease in the safety factor of small-span bridges [23]. Therefore, this study focuses on investigating 8 m RC plate beams specifically designed for heavy-haul railways.

**Figure 1:**
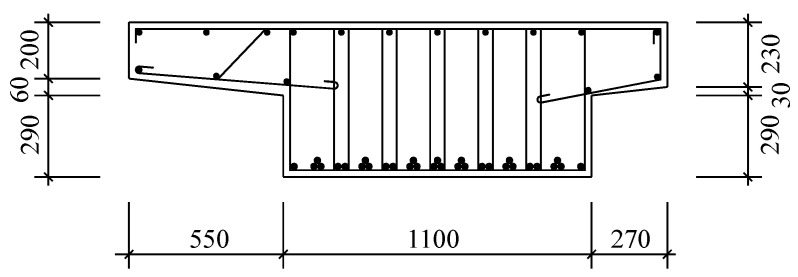
Geometric dimensions and reinforcement of mid-span section of plate beam.

The plate beam has a span of 8 m, a height of 0.55 m, and a width of 1.92 m. The geometric dimensions and reinforcement of the mid-span section of the plate beam are shown in Figure 1. The concrete grade of the plate beam is 350 (equivalent to the concrete strength grade C33). The diameters of tensile steel bars, compressive steel bars, and stirrups inside the beam are 25 mm, 10 mm, and 8 mm, respectively. The tensile steel bars are 16 Mn steel bars with a yield strength of 345 MPa, while the compressive steel bars and stirrups are A3 steel bars with a yield strength of 235 MPa. The second phase dead load is 39.2 kN/m, and each plate beam bears half of the load. The material parameters used in the fatigue reliability evaluation of plate beams are determined through literature [36].

**Figure 2:**
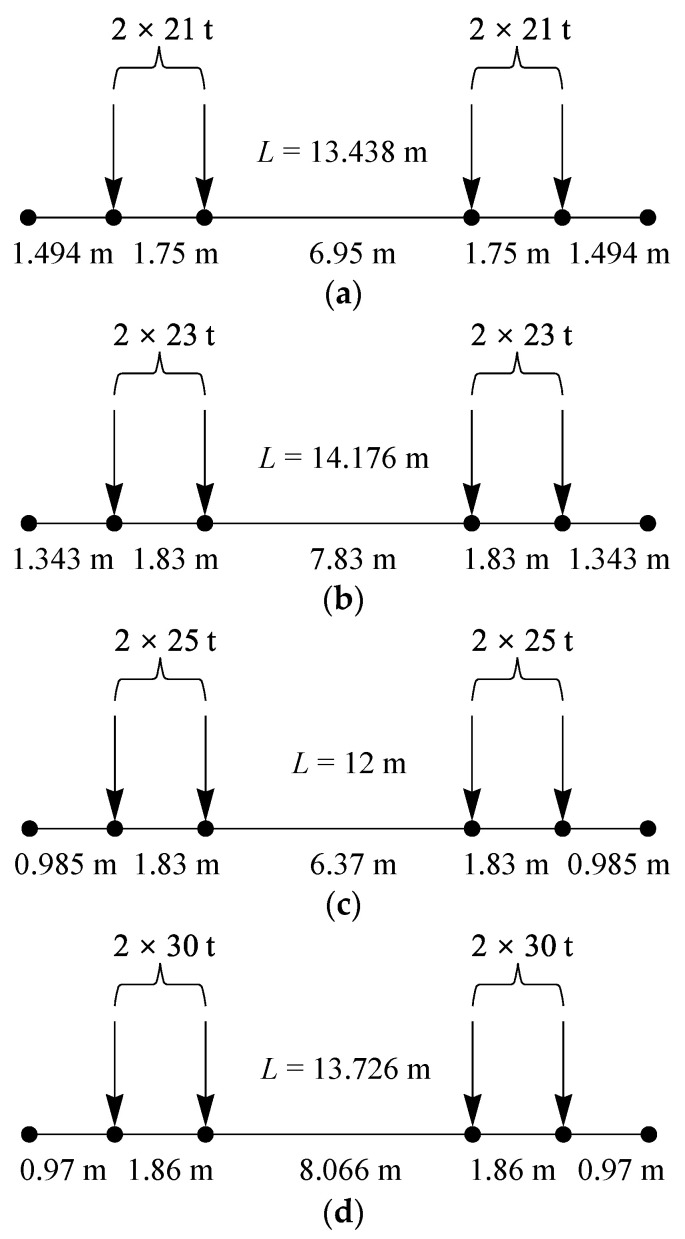
Heavy-haul vehicle load model. (**a**) HV-21; (**b**) HV-23; (**c**) HV-25; (**d**) HV-30.

Before conducting the fatigue reliability assessment, it is necessary to study the load history of heavy-haul railway bridges. For railway bridges, research usually focuses on train formation, axle load, wheelbase, and traffic frequency [37]. Locomotives and heavy-haul vehicles are the basic components of freight trains, and to simplify analysis, the role of locomotives is not considered. The axle loads of heavy-haul vehicles currently in operation in China are 21 t, 23 t, 25 t, and 30 t, respectively. Each type of heavy-haul vehicle is represented as HV-21, HV-23, HV-25, and HV-30. The load model of heavy-haul vehicles with different axle loads is shown in Figure 2, where *L* represents the length of the vehicle.

This study assumes that three different types of freight trains with different traction masses have been operated on the heavy-haul railway so far: ordinary trains, trains with a traction weight of 10,000 t, and trains with a traction weight of 20,000 t. Assume that each type of train consists of heavy-haul vehicle units of the same type. The ordinary train formation is 66 HV-21 cars, represented as FT-N-21; a train with a traction mass of 10,000 t is composed of 108 HV-23 cars, denoted as FT-1-23; a train with a traction mass of 20,000 t is composed of 200 HV-25 or 167 HV-30 cars, respectively, represented as FT-2-25 and FT-2-30. When two different types of trains are operated, each type of train accounts for 50% of the annual traffic volume. Based on the annual traffic volume and operating spectrum of the heavy-haul railway, the equivalent historical operating spectrum of the heavy-haul railway is calculated, as shown in Table 2.

**Figure 3:**
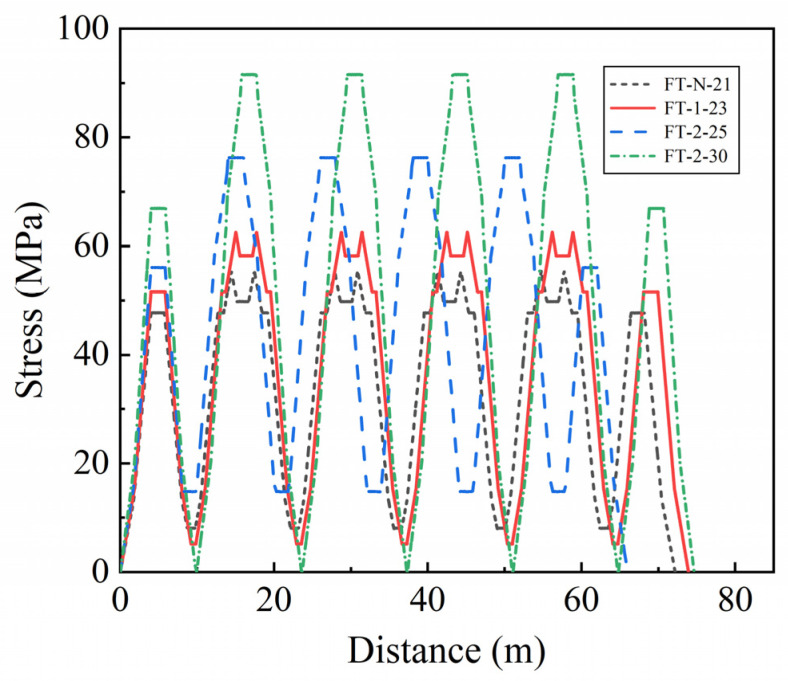
Stress response of tensile steel bar at mid-span beam bottom.

For heavy-haul railway RC beams, it is necessary to obtain the stress response of the tensile steel bars in the mid-span section during the fatigue reliability assessment. This study considers the train wheel axle as a concentrated force and uses the moving load method widely used in bridge fatigue assessment to calculate the bending moment response of the mid-span section of the plate beam when the train passes through the bridge. After obtaining the bending moment of the train passing through the bridge, the stress response of the tensile steel bars in the middle of the span can be calculated using the following equation [36]:

(16) $$\sigma_{s}=n_{s}\frac{My}{I}$$

where *n*_s_ is the ratio of the elastic modulus of the tensile steel bar to the concrete; *M* is the bending moment response at the mid-span of the beam; *y* is the distance from the position of the tensile reinforcement to the neutral axis of the mid-span section; *I* is the moment of inertia of the mid-span section. The dynamic coefficient must be applied to the train load in the moving load method to account for the dynamic effects of moving trains. When the randomness of the dynamic coefficient is not considered, the value of the dynamic coefficient 1 + *μ* is 1.3158 for a simply supported beam with a span of 8 m [38]. Considering the dynamic coefficient of train load can make the calculation results reliable and ensure the fatigue safety of bridges [39]. When the randomness of parameters is not considered and the number of train formations for all four types is 5, the stress response of the tensile steel bars at the bottom of the mid-span section beam is shown in Figure 3.

Cui et al. [18] removed the steel bars from an 8 m span RC beam on the Daqin Heavy Haul Railway and conducted a large number of constant amplitude fatigue tests. The fatigue test data was linearly regressed to obtain the S-N curve suitable for the steel bars in the RC beam of the heavy-haul railway, which can be expressed as:

(17) lgN=17.1778−4.8507lg(Δσ)

### 4.2. Random Variables and Their Probability Distributions

The randomness of train loads has a significant impact on the fatigue reliability of heavy-haul bridges. The train load can be determined by the train axle load *T* and the dynamic coefficient *μ* calculation. Taking *T* and *μ* as random variables, a series of sample combinations can be generated to calculate the train load by sampling them with the Monte Carlo method. By employing the moving load method for stress response calculation of tensile steel bars in RC plate beams, and integrating the rain flow counting technique to determine the equivalent stress range of the steel bars, we can express the equivalent stress range *S*_re_ as follows:

(18) $$S_{\mathrm{re}}=f_{\mathrm{MR}}(T_{1},T_{2},\cdots T_{s},\mu_{1},\mu_{2}\cdots \mu_{s})$$

where *s* represents the total number of wheelsets under different train formations, and $f_{\mathrm{MR}}(\;\cdot \;)$ represents the joint algorithm of the moving load method and rain flow counting method (MLM-RFC).

In the fatigue reliability analysis of RC beams on heavy-haul railways, the train axle load *T* follows a normal distribution with a mean of 1.0179*T* and a standard deviation of 0.0657*T*; the dynamic coefficient *μ* obeys a normal distribution with a mean of 0.15 and a standard deviation of 0.06 [40]. When using the Monte Carlo method to obtain the first four moments of random variables, the data tend to stabilize when the sampling frequency is more than 15,000 times [41]. Therefore, a Monte Carlo method with a sample size of 20,000 was used, which involves conducting 20,000 MLM-RFC calculations to obtain the first four moments of the equivalent stress range *S*_re_ of the tensile steel bars and the corresponding number of cycles, as shown in Table 3.

The existing research results indicate that the critical fatigue damage *D*_c_ of steel bars during fatigue fracture has a certain discrete type, which can be represented by a lognormal distribution with a mean of 1 and a standard deviation of 0.3 [42]. Therefore, the skewness and kurtosis of the random variable *D*_c_ are 0.9270 and 4.6729, respectively. The fatigue performance constant *C* can be represented by a lognormal distribution with a mean of 1.0*C* and a standard deviation of 0.4*C* [32]. Therefore, the mean value, standard deviation, skewness and kurtosis of fatigue performance constant *C* in this paper are 1.5059 × 10^17^, 6.0237 × 10^16^, 1.2640 and 6.1846, respectively. The equivalent stress range *S*_re_ generated by different types of trains passing through bridges is a random variable with an unknown probability distribution. The first four moments of *S*_re_ are shown in Table 3. Utilizing the statistical characteristics of each random variable, this study employs a 5-point estimation on the standard normal space from Table 1 and computes the corresponding random variable’s original space 5-point estimation value through fourth-order moment inverse normal transformation [33], as presented in Table 4.

## 5. Results and Discussion

### 5.1. Fatigue Reliability Evaluation

**Figure 4:**
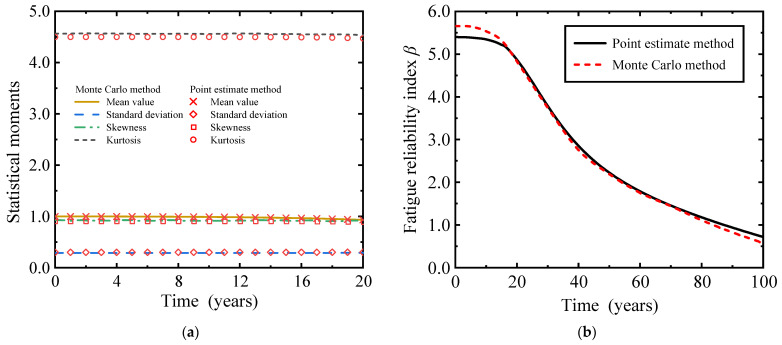
Comparison of results between point estimation method and Monte Carlo method. (**a**) First four moments; (**b**) reliability index.

By employing the point estimate method to assess the fatigue reliability of RC beams in heavy-haul railway applications, in conjunction with the historical equivalent operating spectrum provided in Table 2, we computed the first four moments of the fatigue function for RC slab beams during their initial 20 years of service on heavy-haul railways. The accuracy of our calculations was verified using a Monte Carlo simulation approach with a sample size of 10^7^, as depicted in Figure 4. Among them, the equivalent stress range with unknown probability distribution is sampled by Pearson system random number through the first four moments. As can be observed from Figure 4a, in the first 20 years of heavy-haul railway operation, compared with the results calculated by the Monte Carlo method, the maximum relative errors of the mean, standard deviation, skewness, and kurtosis of the performance functions calculated by the point estimate method were 0.042%, 0.089%, 1.858%, and 1.554%, respectively. In addition, the computational efficiency of the point estimate method is 2 to 3 orders of magnitude higher than that of the Monte Carlo method, indicating that the point estimation method used in this paper has both high accuracy and computational efficiency. It can be observed from Figure 4b that the point estimate method and Monte Carlo method are in good agreement to calculate the fatigue reliability of 8 m plate beams of heavy-haul railway, which proves the accuracy of the proposed method.

**Figure 5:**
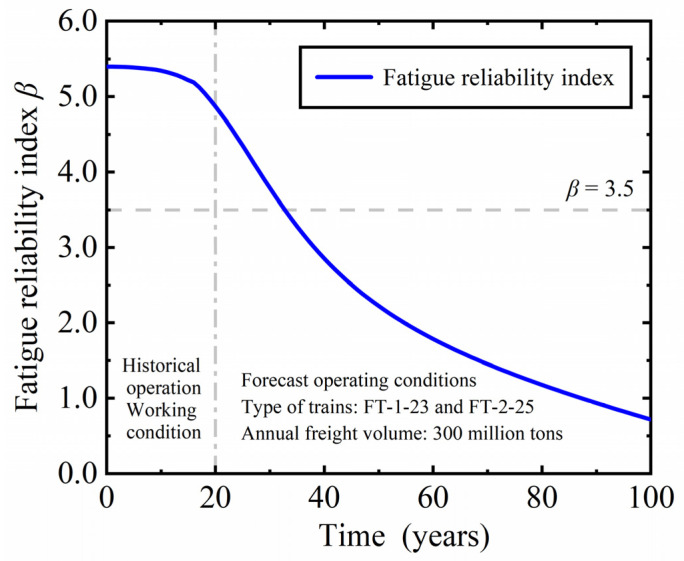
Fatigue reliability index of plate beams changing with service time.

According to the equivalent historical traffic volume of heavy-haul railways given in Table 2, and assuming that from the 21st year of operation to the 100th year, the operating train types are FT-1-23 and FT-2-25, with an annual traffic volume of 300 million tons, remaining unchanged. Figure 5 shows the fatigue reliability indicators of plate beams with changes in service time since the operation of heavy-haul railways. With the continuous increase in the number of overloaded railway trains, the fatigue reliability index of plate beams continues to decline. In the early stages of heavy-haul railway operation, the fatigue reliability index decreased slowly. With the continuous increase in annual traffic volume and the operation of FT-1-23 and FT-2-25, the decrease in fatigue reliability index increased rapidly. After 20 years of operation, the fatigue reliability index of the plate beam decreased from 5.40 to 4.87. Starting from the 7th and 18th year of operation, the annual transportation volume of heavy-haul railways has exceeded 100 million tons and 300 million tons, respectively, with corresponding fatigue reliability indicators of 5.37 and 5.05. The fatigue target reliability index of railway bridge structures specified in the specification is 3.5 [43], and the corresponding failure probability is 2.3263 × 10^−4^. When the heavy-haul railway is in operation until the 33rd year, the fatigue reliability index of the plate beam decreases to below 3.5, and currently, it is necessary to strengthen the maintenance and repair work of the bridge.

### 5.2. Influence of Annual Traffic Volume and Axle Weight of Trains

To study the influence of heavy-haul railway annual traffic volume on the fatigue reliability of RC plate beams, the future traffic volume under different annual traffic load conditions was predicted, as shown in Table 5. The forecast conditions assume that from the 21st to the 100th year of operation, trains FT-1-23 and FT-2-25 will operate, and the annual volume and type of trains will remain the same each year under the same operating conditions.

**Figure 6:**
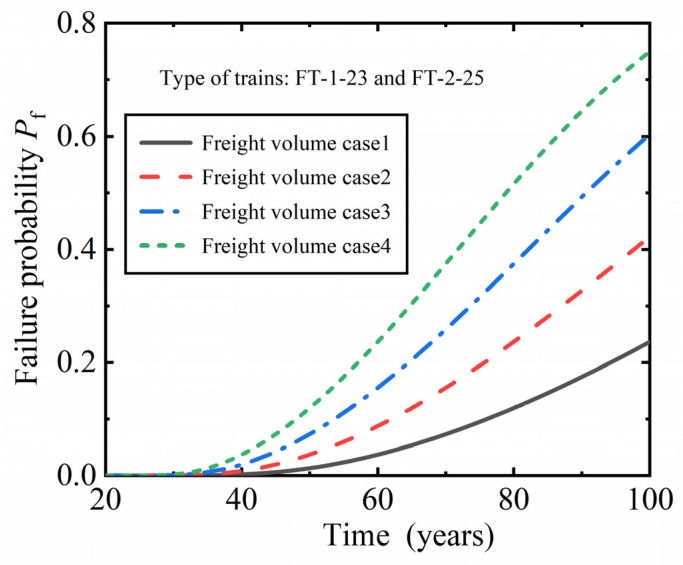
The influence of annual freight volume on the fatigue failure probability of plate beams.

Figure 6 shows the fatigue failure probability of the plate beam with changes in service time under four different traffic conditions. As the service time of the plate beam increases, the probability of failure increases. When the heavy-haul railway operates for the same time, the fatigue failure probability of plate beam increases with the increase in annual traffic volume. Under four different traffic conditions, the fatigue failure probability of the plate beam is greater than 2.3263 × 10^−4^ for heavy-haul railways operating until the 33rd, 30th, 28th, and 27th years, respectively, exceeding the limit specified in the specification; i.e., the fatigue reliability index is less than 3.5.

To study the effect of axle load of heavy-haul railway trains on the fatigue reliability of RC plate beams, the traffic volume under different axle load conditions of trains in the future was predicted, as shown in Table 6. The predicted operating conditions assume an annual traffic volume of 300 million tons from the 21th to the 100th year of operation, and the annual traffic volume and train types operating under the same operating conditions remain unchanged.

**Figure 7:**
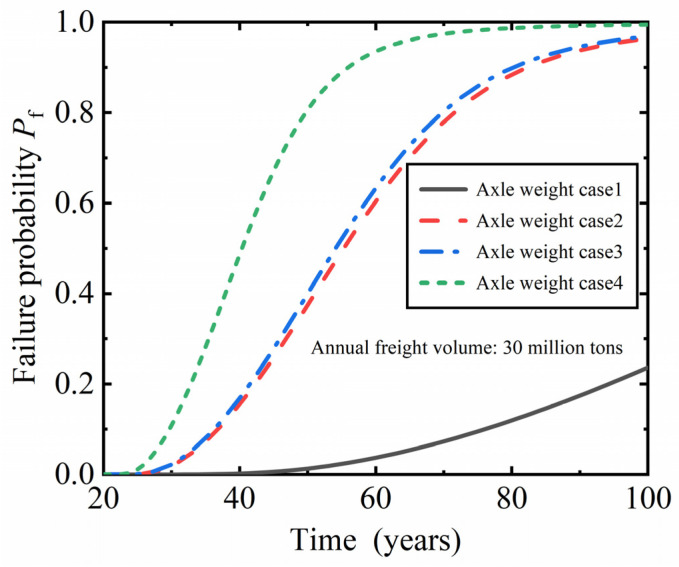
The influence of axle weight on fatigue failure probability of plate beams.

Figure 7 shows the fatigue failure probability of the plate beam with changes in service time under four axle load conditions. As the service time of the plate beam increases, the probability of failure increases. When the heavy-haul railway operates for the same time, the increase in axle load of the operating train increases the probability of fatigue failure of the plate beam. When the axle load of the operating train increases from 23 t to 25 t (axle load conditions 2 to 3), the probability of fatigue failure slightly increases. This is caused by the increase in train axle load and number of formations, but when transporting goods of the same weight, the number of FT-1-23 trains is about 2.1 times that of FT-2-25; thus, the increase in fatigue failure probability is relatively small. As the axle load of operating trains increases from 25 t to 30 t (axle load conditions 1 to 2, 3 to 4), the probability of fatigue failure significantly increases. The main reasons for this phenomenon are: (1) the increase in axle load; (2) the length between the second and third axes of HV-30 is greater than the span of the plate beam, causing the lower limit of fatigue load to decrease to 0, thus increasing the fatigue stress range of the steel bars. This is similar to the research conclusion in the existing literature [1].

The fatigue failure probability of plate beams is greater than 2.3263 × 10^−4^ when the heavy-haul railway is operated to the 33rd, 24th, 24th and 23rd years, respectively, under four axle load conditions. When the number of 30 t trains accounts for 50% and 100% of the total number of operating trains, the fatigue reliability index of the plate beam decreases to below 3.5 in the fourth and third years after the operation of 30 t axle load trains, which is lower than the standard limit value. Therefore, the operation of a 30 t axle load train will seriously reduce the fatigue performance of the plate beam.

## 6. Conclusions

This study established the fatigue performance function of RC beams for heavy-haul railways and developed a fatigue reliability evaluation method for RC beams for heavy-haul railways based on the point estimate method. Taking an 8 m RC plate beam of a heavy-haul railway as an example, the train load model of the heavy-haul railway was analyzed. The train axle load and dynamic coefficient were used as random variables to obtain the first four moments of equivalent stress range, and the operating spectrum of the heavy-haul railway was calculated. On this basis, the fatigue reliability assessment was conducted on the 8 m plate beam. The conclusion drawn is summarized as follows:

1. The fatigue reliability evaluation method for heavy-haul railway RC beams based on the point estimate method can efficiently and accurately evaluate the fatigue reliability of heavy-haul railway RC beams, and the calculation efficiency is improved by 2 to 3 orders of magnitude compared to the Monte Carlo method.
2. The fatigue performance of the 8 m plate beam during the initial two decades of heavy-haul railway operation exhibited favorable characteristics, with a probability of fatigue failure that remained below the specified limit outlined in the specifications. Starting from the 21st year of operation, the annual transportation volume is 300 million tons, and the axle loads of operating trains are 23 t and 25 t, which remain unchanged every year. When operating until the 33rd year, the fatigue reliability index of the plate beam decreases to below 3.5.
3. The annual transportation volume has a significant impact on the fatigue reliability of plate beams. Starting from the 21st year of operation, the axle loads of the operating trains are 23 t and 25 t, and remain unchanged every year. When the annual transportation volumes are 300 million tons, 400 million tons, 500 million tons, and 600 million tons, respectively, the fatigue reliability index of the plate beam decreases to below 3.5 in the 33rd, 30th, 28th, and 27th years of operation.
4. Running a 30 t axle load train will cause a serious decrease in the fatigue reliability of the plate beams. Starting from the 21st year of operation, when the number of 30 t trains accounts for 50% and 100% of the total number of operating trains, the fatigue reliability index of the plate beam decreases to below 3.5 in the fourth and third years after the operation of 30 t axle load trains.

In conclusion, the fatigue reliability of reinforced concrete bridges for heavy-haul railways is studied in detail in this paper, and the research results are expected to provide an important basis for the design and maintenance of reinforced concrete bridges for heavy-haul railways in the future. Future research will focus on the fatigue reliability analysis method of RC bridge, which combines nonlinear finite element analysis with the point estimate method, to solve the problem of the too-low computational efficiency generated by nonlinear numerical simulation, and to balance the computational accuracy and computational efficiency in the structural reliability analysis.

## Figures and Tables

**Table 1:** Estimation points and corresponding weights of 5-point estimation in standard normal space.

Integration Point	Weight Values
*u*_r,1_ = −*u*_r,5_ = −2.85697001	*P*_r,1_ = *P*_r,5_ = 1.12574113 × 10^−2^
*u*_r,2_ = −*u*_r,4_ = −1.35562618	*P*_r,2_ = *P*_r,4_ = 2.22075922 × 10^−1^
*u*_r,3_ = 0	*P*_r,3_ = 5.33333333 × 10^−1^

**Table 2:** Equivalent historical operating spectrum of heavy-haul railway.

Time (Year)	Annual Volume of Traffic (10,000 Tons)	Train Frequency		
		FT-N-21	FT-1-23	FT-2-25
1	548	1361	-	-
2	1655	4111	-	-
3	3254	8083	-	-
4	5439	13,510	-	-
5	7470	18,555	-	-
6	9292	23,080	-	-
7	11,106	27,586	-	-
8	10,133	25,169	-	-
9	11,437	28,408	-	-
10	14,647	18,191	9687	-
11	15,900	19,747	10,516	-
12	16,700	20,740	11,045	-
13	19,760	24,540	13,069	-
14	23,957	29,753	15,845	-
15	25,300	31,421	16,733	-
16	21,500	26,701	14,219	-
17	27,200	-	17,989	8500
18	30,471	-	20,513	9522
19	31,600	-	20,899	9875
20	30,450	-	20,139	9516

**Table 3:** The statistical moment of equivalent stress range and the corresponding number of cycles.

Equivalent Stress Range	Train Type	Number of Cycles	Mean Value	Standard Deviation	Skewness	Kurtosis
*S*_re,21_	FT-N-21	132	38.2666	0.2538	0.0531	3.0417
*S*_re,23_	FT-1-23	216	45.7364	0.2265	0.0160	2.9811
*S*_re,25_	FT-2-25	201	56.8803	0.2244	−0.0111	3.0650
*S*_re,30_	FT-2-30	168	82.8756	0.3028	−0.0111	3.0058

**Table 4:** 5-point inverse normal transformation value of random variables.

Random Variable	Point 1	Point 2	Point 3	Point 4	Point 5
*D*_c_	0.397055	0.643907	0.957565	1.427190	2.210805
*C*	3.795159 × 10^16^	8.315966 × 10^16^	1.397218 × 10^17^	2.362295 × 10^17^	4.189110 × 10^17^
*S*_re,21_	37.551659	37.925055	38.264375	38.611873	39.013418
*S*_re,23_	45.096345	45.429573	45.735793	46.044244	46.385149
*S*_re,25_	56.227534	56.576687	56.880709	57.183228	57.527214
*S*_re,30_	82.005459	82.464761	82.876159	83.285502	83.737728

**Table 5:** Forecast traffic volume under different annual traffic conditions.

Operating Condition	Annual Volume of Traffic (10,000 Tons)	Train Frequency	
		FT-1-23	FT-2-25
1	30,000	19,841	9375
2	40,000	26,455	12,500
3	50,000	33,069	15,625
4	60,000	39,683	18,750

**Table 6:** The predicted traffic volume of trains under different axle load conditions.

Operating Condition	Train Frequency		
	FT-1-23	FT-2-25	FT-2-30
1	19,841	9375	-
2	19,841	-	9356
3	-	9375	9356
4	-	-	18,713

## Data Availability

Data are contained within the article.
